# Avoidance behaviour in laboratory house mice (*Musmusculus*) and Norway rats (*Rattus norvegicus*) towards predator odours

**DOI:** 10.1371/journal.pone.0245441

**Published:** 2021-01-20

**Authors:** Luciana B. Adduci, Vanina A. León, Annika Schlötelburg, María Busch, Jimena Fraschina

**Affiliations:** 1 Facultad de Ciencias Exactas y Naturales, Facultad de Ciencias Exactas y Naturales, Departamento de Ecología, Genética y Evolución, Universidad de Buenos Aires and Instituto de Ecología, Genética y Evolución de Buenos Aires (IEGEBA), UBA-CONICET, Universidad de Buenos Aires, Intendente Güiraldes 2160—Ciudad Universitaria—C1428EGA, Ciudad Autónoma de Buenos Aires, Argentina; 2 Division of Land Use Systems, Humboldt-University of Berlin, Faculty of Life Science, Institute of Agriculture and Horticulture, Albrecht-Thaer-Weg, Berlin, Germany; University of Sydney, AUSTRALIA

## Abstract

*Mus musculus* and *Rattus* sp. are considered pest species because they reach high densities in urban areas, crop fields and food storage and productive systems such as breeding farms and orchards. Their control relies mainly on rodenticide application, but the effectiveness of this application is reduced due to behavioural responses and resistance. Novel methods are based on the use of chemical signals as odours that may be attractants, repellents or may reduce the reproductive success of pest species. The aim of this paper is to study the aversive effect of TMT, cat urine and cat body odour on predator-inexperienced *Mus musculus* and *Rattus norvegicus* under laboratory conditions. The experimental apparatus comprised three boxes connected by PVC pipes in a linear arrangement. In lateral boxes, odour sources or distilled water were introduced, while animals were placed in the central box at the beginning of the experiment. Rats showed freezing behaviour, reduced visits in the presence of TMT and cat fur. Mice reduced their visits with cat body and cat urine. This study provides evidence of the usefulness of using fear responses as a way to control rodent pests, which must be adapted to the environment and species to be applied.

## 1. Introduction

Human activity causes environmental changes that have large effects on many animal species. While in many cases these effects are negative, many rodent species benefit from anthropogenic changes because of an increase in food sources or refuges (in agricultural or urban areas) or a decrease in predator density [1]. These species may reach pest densities in anthropized habitats, causing several damages through the consumption of food, contamination, damaging building structures, reducing distribution of some endangered species and transmitting diseases to both humans and domestic animals [2–6].

Among the main rodent pest species, *Mus musculus* and *Rattus* sp. are cosmopolitan species reaching high densities in urban areas, crop fields, and food storage and productive systems such as breeding farms and orchards [7]. Their control relies mainly on the application of anticoagulant rodenticides, but it involves some environmental risk by poisoning of non-target species [8,9]. Furthermore, the effectiveness of rodenticide application can decrease over time because rodent populations can develop aversive behaviours and genetic resistance [10].

Recent advances in ecological research and analytical technology have led to novel methods that use chemical signals to create effective attractants and repellents for pest species [11]. This potential use of chemical signals for managing pest species is based on the behavioural and physiological responses to intraspecific or predator odours [12–15], to plant secondary metabolites and to toxic substances [16,17].

Many studies explored the effect of odours on reproduction [13], aversive behaviours or food intake [16] and suggested their potential use as alternative methods for rodent control [18]. <Predator odours, such as feline urine and 2,3,5-trimethylthiazoline (TMT), which is a component of red fox (*Vulpes vulpes*) faeces, have an aversive and reproductive effect on rodents[19,20]. Cat collars, cloth rubbed on cats and cat fur were also demonstrated to have an aversive effect on rats [19,21]. The response to predator odours may elicit innate reactions in rodents, including stereotyped avoidance behaviours, but the future fear response can be modulated by experience [18]. Behavioural responses associated with the perception of predation risk were considered avoidance to move towards the odour source, short permanence in the vicinity of the odour source, and fear and alert responses such as freezing, sniffing, escape attempts and exploration [21–24]. Some authors consider grooming to be a non-defensive behaviour [19,21], while others assume that it is a response to stress stimuli that reflects the process of dearousal due to the termination of a stressful situation [25,26].

In rural habitats of central Argentina, rats and domestic mice reach high densities on breeding farms, where they cause economic damage through food consumption and contamination, damaging building structures, and, in poultry farms, kill chicks [27,28]. They can also transmit diseases to both humans and domestic animals [2–4,28]. In spite of rodenticide application, most poultry farms are infested with rodents [27], probably because of recolonization after control and the presence of individuals with low sensitivity to anticoagulants [29,30]. In previous works, we explored the potential of different odours as alternative methods for their control in both laboratory and semicaptivity conditions. In laboratory conditions, the reproductive success of *M*. *musculus* females was affected by unfamiliar males, cat urine and TMT odours [31], while domestic and Geoffroy’s cat odours did not produce avoidance in wild *M*. *musculus* in semicaptivity conditions [28].

The aim of this paper is to study the aversive effect of TMT, cat urine and cat body odour on predator-inexperienced *M*. *musculus* and *R*. *norvegicus* individuals under laboratory conditions. Our hypothesis was that individuals of both species will avoid TMT, cat urine or cat body odours and will display “alert behaviours” in their presence.

## 2 Materials and methods

The procedures conducted in this work were approved by the Institutional Comittee for Care and Use of laboratory animals (CICUAL, FCEN, Universidad de Buenos Aires), protocol number 88.

### 2.1. Subjects

Many studies have used laboratory albino animals [21,23,32,33] to assess the effect of chemical signals on rodent behaviour. The use of laboratory animals has the advantage of the facility to obtain sufficient animals of both sexes, with known age, similar genetic composition and life histories, thus decreasing potential heterogeneities in responses. On the other hand, the use of laboratory animals to preselect potential products for use in rodent control may reduce the number of wild animals used in experiments, in accordance with EU Directive 2010/63.48. Although laboratory animals have been isolated from environmental cues for many generations, they show generalized responses to odours from historical predators [34]. In consequence we used, as a first step prior to experiments with wild animals, laboratory mice and rats from domestic strains.

The subjects (n = 128) were male and female *M*. *musculus* (CrlFcen: CF 1 mice) and *R*. *norvegicus* (HsdFcen:WI) obtained from the breeding colony of the animal husbandry unit of the Facultad de Ciencias Exactas y Naturales, Universidad de Buenos Aires, Argentina. Both males and females were sexually inexperienced and between 9 and 10 weeks old. Prior to the experiments, rodents were housed individually for 7 days for acclimatization in metal cages (41 x 36 x 17 cm) with softwood shavings, cotton and cardboard tubes as nesting material, and food (commercial food pellets, Cooperación ACA Nutrición Animal) and water *ad libitum*. They were kept at a temperature of 23°C on a 12:12 h light–dark cycle.

### 2.2. Odour sources

The tested odours included TMT, cat urine, cat body odour and distilled water as a control. TMT (97.5%) was obtained from SIGMA ALDRICH® (now Merck KGaA, Darmstadt, Germany). Because of logistic reasons, we could use only urine from one domestic, castrated female cat, which frequently hunts wild rodents and was fed with meat. This cat was trained to urinate in a container allowing urine collection. For the cat body odour, a piece of cloth (5 x 5 cm) was rubbed vigorously against male and female domestic cats (n = 4) for 5 min, according to Muñoz Abellán et al. and File et al [35,36]. Urine samples and cloth pieces were frozen (-18°C) until experiments were performed and were unfrozen at least 30 min before they began. For liquid odours and distilled water, a volume of 1 ml was applied to a tissue paper. The use of distilled water as a control was decided according to Fendt et al., [20] and Horii et al., [23].

### 2.3. Apparatus

The test apparatus comprised three transparent plastic boxes (19 x 30 x 23 cm) where both mice and rats could stand on their hind legs, connected by two opaque PVC pipes (50 cm length; 7 cm diameter) in a linear arrangement. This device was placed in a room with evenly distributed light of 40 watt intensity to avoid differences in light intensity among boxes.

### 2.4. General procedure

The experiments were conducted in the facilities of the Facultad de Ciencias Agrarias, Universidad Nacional de Lomas de Zamora, Argentina (34.77° South, 58.45° West), from November 2017 to August 2018.

To allow habituation to the testing environment all animals received a daily 10-min habituation session over four consecutive days [37]. They were moved from the animal room to the test apparatus with blank stimuli (Petri dishes with tissue papers without odour). Odour exposure tests began on the fifth day. After tested, animals did not return to the vivarium where other animals waited to be taken to the experimental room.

On the exposure days, we placed Petri dishes covered with metal mesh (to prevent direct contact of animals with the odour source) in the right (odour source) and left (distilled water as control) boxes. We decided to maintain the box with odour (right box) along the different trials to prevent remaining odours from affecting the results, even though the experimental device was thoroughly cleaned after each trial. Rodents were individually placed in the central box of the experimental apparatus. We also conducted trials with distilled water in both lateral boxes. We renewed the tissue papers every three trials while the cloths with cat body odour were renewed after every trial. The test apparatus, the petri dishes and the metal mesh were vigorously cleaned at the end of each observation with a 70% ethanol solution. We assessed the effect of each different odour on different days, and after the sessions of one day, the test apparatus was intensely cleaned.

Animals were randomly assigned to the type of trial (treatment): TMT- Control (TMT), Cat Urine- Control (Cat Urine), Cat body- Control (Cat Body) and Control- Control (Control). Each odour was tested with 16 house mice (8 females, 8 males) and 16 rats (8 females, 8 males). All sessions started with the rodent placed at the central compartment of the experimental device. Behavioural responses were recorded over 10 min by two observers placed 2 meters from the experimental apparatus. We did not observe behaviours suggesting that animals were influenced by the presence of observers. We recorded at each second the location of the individual when the entire body was inside the compartment: central, odour, control box or connecting pipes (left and right), and the occurrence of the following behaviours: freezing, sniffing and grooming.

### 2.5. Data analyses

We conducted analyses separately for each species using the statistical program R (version 3.5.1, RCore Team 2018). To assess the effect of odours on avoidance behaviour, we compared the proportion of individuals who visited the odour box at least once for treatments with odours with respect to the Control by means of a one-sided test of difference between proportions [38]. We constructed models in which we considered as response variables the total number of visits to the odour box (*Visits*) and the number of seconds (out of 600) in which the animal stayed at the odour compartment (*Duration*), considering the total time as a sum of the duration in each visit.

The effect of odours on “alert behaviours” was assessed by the frequency (time intervals in which the behaviour was displayed with respect to the total time of observation) of sniffing (*Sniffing*) and grooming (*Grooming*). We did not conduct models for freezing behaviour because it was displayed by few individuals. We examined the effect of explanatory variables using generalized linear mixed models (GLMM) with the R package “lme4” [39], Matrix [40] and GLMM TMB [41]. Models were fitted by maximum likelihood (Laplace approximation) and selected according to AIC values.

For *Visits*, we first ran models including sex and treatment and their interactions as fixed explanatory variables. The time of the experiment was included as a random factor due to logistic limitations because we were not able to perform all replicates at the same time. For both species, we found significant interactions between the effect of treatment and sex, and in consequence, we conducted Tukey Multiple comparisons, with–“emmeans” package [42]. The best models for mice were ZAP- Zero-altered Poisson models. For rats, we adjusted a GLMM with a Poisson distribution of errors and a log-log link function.

For the variable *Duration*, we adjusted GLMM with a binomial distribution of errors. For both species, the models included the sex, treatment and their interactions as fixed explanatory variables and the time of the experiment and the individual (to avoid overdispersion) as random factors [43]. For mice, we did not find a significant interaction between the treatment and sex, while for rats there was a significant interaction, and in consequence we conducted Tukey multiple comparisons to test for differences among treatments according to sex with the emmeans package [42].

For the variables *Sniffing* and *Grooming* we ran GLMM including the sex and treatment (TMT, Cat Urine, Cat Body and Control) as fixed factors and the individual as a random factor. We assumed a hurdle Poisson distribution of errors. In the GLMM we compared odour treatments with the Control (intercept).

## 3. Results

### 3.1. Visits

The number of mice that visited at least once the odour box did not differ significantly (p>0.050 in all cases) between odour treatments and the Control. For rats, fewer animals visited the odour box for the Cat Body treatment than the Control treatment (p = 0.039; Table 1).

**Table 1:** Number of animals that visited the odour box at least once according to the species and treatment.

	Treatment			
	TMT	Cat Urine	Cat Body	Control
*Mus musculus*	7	8	7	10
*Rattus norvegicus*	12	16	11*	15

* indicates p value<0.05.

**Fig 1:**
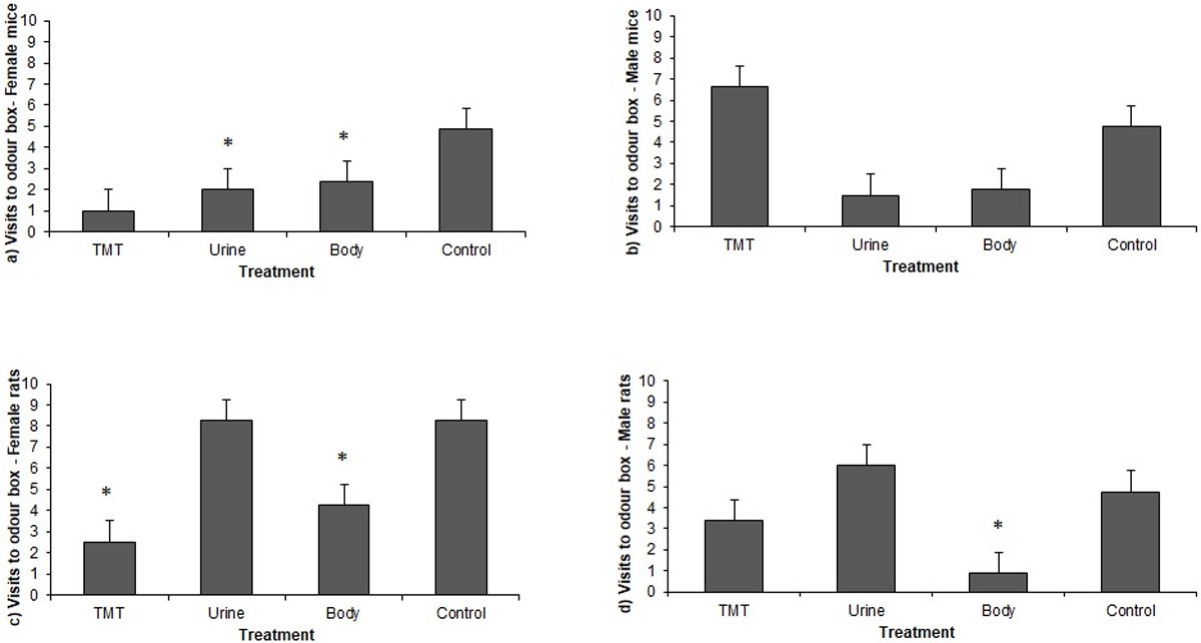
Mean number of visits (+SE) to the odour box according to the treatment for a) female *Mus musculus*, b) male *M*. *musculus*, c) female *Rattus norvegicus*, d) male *R*. *norvegicus*(* indicates p-value<0.05).

The number of visits of female mice to the odour box was lower for the Cat Body and Cat Urine treatments than for the Control treatment (p = 0.00314, p = 0.012, respectively, Tukey test, df = 50), while for male mice there were no significant differences between odour treatments with respect to the Control in the number of visits to the odour box (Fig 1 and Tables A.2 and A.3). Female rats visited the odour box less frequently in the Cat Body and TMT treatments than in the Control treatment (p = 0.0358 and p< 0.001 respectively, Tukey test, df = 55, Fig 1 and Tables A.4 and A.5). Male rats visited the odour box less frequently in the Cat Body than in the Control treatment (p = 0.001, Fig 1 and Tables A.4 and A.5).

### 3.2. Duration

**Fig 2:**
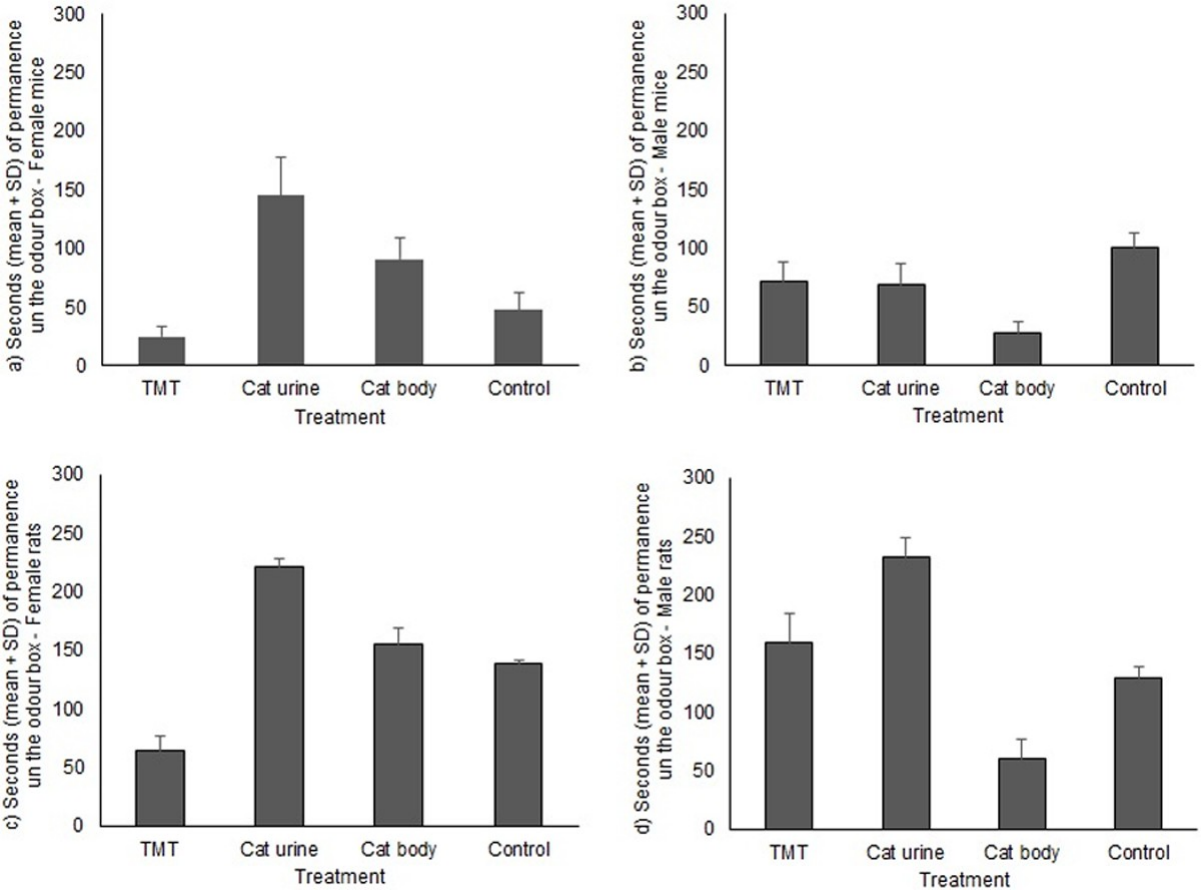
Mean (+SE) duration (in seconds) of the permanence in the box odour according to the treatment for a) female *Mus musculus*, b) male *M*. *musculus*, c) female *Rattus norvegicus*., d) male *R*. *norvegicus*(* indicates p-value<0.05).

For both species the best model for the duration of visits to the odour box according to treatment was the null model (lowest AIC), (Fig 2 and Tables A.6 and A.7).

### 3.3. Behaviours

From all possible behavioural responses, we only observed rats freezing, sniffing and grooming and mice sniffing and grooming, probably because of the confined conditions within the test chamber [20], especially for rats. Freezing was only observed in rats in the TMT treatment; one female in the control box and one female and one male in the central box.

#### 3.3.1. Sniffing

**Fig 3:**
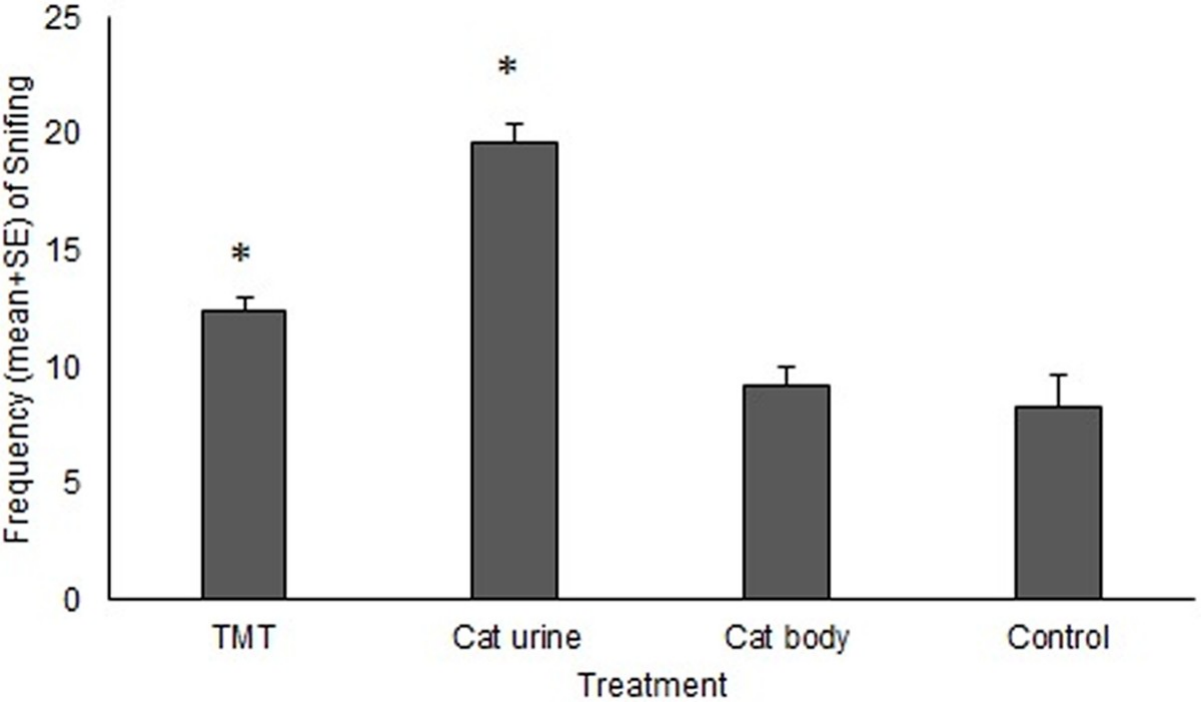
Mean frequency of *Sniffing* in the different treatments for rats.

The mean frecuency of sniffing was 4.54±1.45 for mice and 12.27±2.19 for rats. Mice did not show a treatment effect on the frequency of sniffing neither an effect of sex. For rats there was an effect of treatment and sex, but not an interaction between them. Sniffing was more frequent in both Cat Urine and TMT treatments with respect to the Control (p<0.0001 and p = 0.01, respectively, df = 55 Fig 3). Females showed a higher frequency of sniffing than males (14,9±1.50 versus 9.45±1.47, p<0.01, Tukey test, df = 55).

#### 3.3.2. Grooming

In comparison, rats display more frequently the grooming behavior than mice (mean grooming = 0.72±0.05 and 0.24±0.11, respectively, p<0.0001). Both species showed a lower frequency of grooming than sniffing (mean sniffing = 4.54±0.57 and 12.34±1.54, for mice and rats, respectively). For mice, the analysis could not be done due to the excess of zeros and the low frequency in which they display this behaviour. For rats, the best model did not include the effect of the treatment (AIC null model: 152,61, df = 4, AIC model: 164,9, df = 14).

## 4. Discussion

We investigated whether cat urine, cat body or TMT odours elicited avoidance and alert behaviours in laboratory mice and rats. We considered the number and duration of visits to odour boxes in relation to control boxes with distilled water and alert behaviours as sniffing, grooming and freezing as evidence of avoidance to an odour source.

We found evidence of aversive behaviour in rats to TMT and cat body odours. TMT was the only odour that caused freezing behaviour in this species and its effect was also expressed in a lower number of female’s visits to the odour box and a higher frequency of sniffing of both sexes in this treatment with respect to the Control. The effect of Cat body odour was expressed in a lower proportion of individuals who visited the odour box than in the Control treatment and in a lower number of visits of both sexes to the odour box. The only significant effect of Cat Urine in rats was a higher frequency of sniffing with respect to the control treatment. Female mice showed an aversive behaviour to Cat body and Cat Urine odours, reducing the number of visits to the odour box in these treatments, while males did not show significant effects.

There were no effects of odour treatments on the duration of visits for any species, suggesting that the aversive effect is not maintained once the rodent registers that there is no predator in the box, and in consequence it ignores the odour. This behaviour shows that the innate response to odours may be modified according to experience, as suggested by Bedoya-Pérez et al [18].

Differences in the effect of odours between rats and male mice may be related to differences in risk assessment behaviour, while mice actively approached and investigated possible dangers, this behaviour was not observed in rats [32].

The absence of an effect of treatments on the frequency of grooming suggests that this behaviour, in our experimental conditions, was not a response to the odours, according to the idea that it is a non-defensive behaviour. Grooming can occur once the animal considers that the surroundings are safe [44].

Our results must be interpreted taking in account the particular conditions of the experiment, the distance from the central and lateral boxes was only 50 cm, and the total length from the odour to the control box was 130 cm (50 cm of each tube plus 30 cm of the central box). This size may have caused an odour effect in all the apparatus, but we consider that animals reacted identifying the source of odour. We cannot discard, however, an apparatus effect, especially in rats who display freezing behaviour in the TMT treatment. It would have been more adequately to use species specific experimental apparatus, with higher size for rats. According to Blanchard et al. [32], results may also be interpreted taking in account potential effects of the origin (laboratory or wild) and strain of the animals used, as well as species and gender effects. In consequence, when considering the potential use of odours as pest repellents in field conditions, our results must be interpreted taking in account the characteristics of the experiment and the test apparatus, in which the small size allowed animals to explore and discard the presence of any predator. In field conditions, the “uncertainty” may last longer. On the other hand, odours are concentrated in the laboratory while in the field they diffuse more readily.

## 5. Conclusion

In conclusion, we found a potential aversive effect of TMT and cat body odour on rats, and cat body and cat urine in female mice. The effect of TMT was expressed in the number of visits and in the freezing behaviour that was only observed in rats in the TMT treatment. The use of cat body odour as a repellent in field conditions may be limited because it is not volatile and because animals may have an effect near or in contact with the source of odour [45]. Despite the fact that some authors [46,47] consider that TMT lacks some specific qualities of cat body/skin odour, this does not preclude the usefulness of its aversive effect for rodent control because it also has a detrimental effect on mouse reproduction [31]. We expect more effects on rats, which cause more damage in farm buildings than mice, including chicken mortality (Noriega com. pers.).

Our work provides evidence of the usefulness of using fear responses as a way of managing rodent pests, but, as pointed by Bedoya-Pérez et al [18], it is not easy, because anti predator responses are embedded in complex ecological systems and rely on complex contextual clues.

## Appendix

**Table A.2:** Generalized linear mixed model (GLMM) results for the number of visits to the odour box per treatment for mice, considering the experiment as a random effect, with ZAP model (Zero-inflation Poisson distribution). Signif. codes: 0 ‘***’, 0.001 ‘**’, 0.01 ‘*,’ 0.05 ‘.’.

Explanatory variable	Estimate	SE	Z	P-value
Intercept (Control)	2.2772	0.1602	14.217	<2e-16 ***
Cat Body	-0.9671	0.2893	-3.343	0.000830 ***
Cat urine	-1.1627	0.3152	-3.689	0.000225 ***
TMT	-0.9109	0.4018	-2.267	0.023372 *
Sex: Male	-0.4332	0.2286	-1.895	0.058141
Odour Cat Body:SexMale	1.0680	0.4269	2.502	0.012348 *
OlorCat urine:SexMale	0.6849	0.4653	1.472	0.141003
OlorTMT:SexMale	1.4277	0.4549	3.139	0.001697 **

SE = Standard error. Z = parameter estimated. Total number of observations: 64. Df.resid = 50.

**Table A.3:** Tukey comparison for all possible pairs for the model in Table A.2. Signif. codes: 0 ‘***’, 0.001 ‘**’, 0.05 ‘*’.

Contrast	Estimate	SE	df	t.ratio	p.value
Control Female—Cat body Female	0.9671	0.289	50	3.343	0.0314 *
Control Female—Cat urine Female	1.1627	0.315	50	3.689	0.0120 *
Control Female—TMT Female	0.9109	0.402	50	2.267	0.3315
Control Female—Control Male	0.4332	0.229	50	1.895	0.5609
Control Female—Cat body Male	0.3322	0.312	50	1.064	0.9613
Control Female—Cat urine Male	0.9109	0.341	50	2.673	0.1553
Control Female—TMT Male	-0.0836	0.211	50	-0.396	0.9999
Cat body Female—Cat urine Female	0.1956	0.363	50	0.539	0.9994
Cat body Female—TMT Female	-0.0561	0.440	50	-0.128	1.0000
Cat body Female—Control Male	-0.5339	0.291	50	-1.835	0.6002
Cat body Female—Cat body Male	-0.6349	0.360	50	-1.761	0.6482
Cat body Female—Cat urine Male	-0.0561	0.385	50	-0.146	1.0000
Cat body Female—TMT Male	-1.0507	0.277	50	-3.788	0.0090
Cat urine Female—TMT Female	-0.2517	0.458	50	-0.550	0.9993
Cat urine Female—AControl Male	-0.7295	0.317	50	-2.303	0.3123
Cat urine Female—Cat body Male	-0.8304	0.382	50	-2.177	0.3829
Cat urine Female—Cat urine Male	-0.2517	0.405	50	-0.621	0.9984
Cat urine Female—TMT Male	-1.2463	0.304	50	-4.096	0.0036
TMT Female—AControl Male	-0.4778	0.403	50	-1.186	0.9323
TMT Female—Cat body Male	-0.5787	0.456	50	-1.270	0.9055
TMT Female—Cat urine Male	0.0000	0.476	50	0.000	1.0000
TMT Female—TMT Male	-0.9946	0.393	50	-2.529	0.2073
Control Male—Cat body Male	-0.1010	0.314	50	-0.322	1.0000
Control Male—Cat urine Male	0.4778	0.342	50	1.396	0.8548
Control Male—TMT Male	-0.5168	0.213	50	-2.423	0.2531
Cat body Male—Cat urine Male	0.5787	0.403	50	1.436	0.8361
Cat body Male—TMT Male	-0.4158	0.301	50	-1.380	0.8618
Cat urine Male—TMT Male	-0.9946	0.331	50	-3.007	0.0733

**Table A.4:** Generalized linear mixed model (GLMM) results for the number of visits to the odour box per treatment for rats, considering the experiment as a random effect, with Poisson distribution. Signif. codes: 0 ‘***’, 0.001 ‘**’, 0.01 ‘*,’ 0.05 ‘.’.

Explanatory variable	Estimate	SE	Z	P-value
(Intercept)	2.110e+00	1.231e-01	17.143	< 2e-16 ***
Cat Body	-6.633e-01	2.111e-01	-3.142	0.00168 **
Cat urine	6.281e-15	1.741e-01	0.000	1.00000
TMT	-1.194e+00	2.552e-01	-4.678	2.9e-06 ***
Sex: Male	-5.521e-01	2.036e-01	-2.711	0.00671 **
Olor Cat Body: Sex Male	-1.028e+00	4.623e-01	-2.224	0.02612 *
Olor Cat urine: Sex Male	2.336e-01	2.783e-01	0.839	0.40123
Olor TMT: Sex Male	8.522e-01	3.585e-01	2.377	0.01744 *

SE = Standard error. Z = parameter estimated. Total number of observations: 64. Df.resid = 55.

**Table A.5:** Tukey comparison for all possible pairs for the model in Table A.4. Signif. codes: 0 ‘***’, 0.001 ‘**’, 0.05 ‘*’.

Contrast	Estimate	SE	df	z.ratio	p.value
Control Female—Cat Body Female	0.663	0.211	55	3.142	0.0358 *
Control Female—Cat urine Female	0.000	0.174	55	0.000	1.0000
Control Female—TMT Female	1.194	0.255	55	4.678	0.0001 **
Control Female—Control Male	0.552	0.204	55	2.711	0.1191
Control Female—Cat body Male	2.244	0.398	55	5.645	< .0001
Control Female—Cat urine Male	0.318	0.190	55	1.679	0.7012
Control Female—TMT Male	0.894	0.228	55	3.913	0.0023
Cat body Female—Cat urine Female	-0.663	0.211	55	-3.142	0.0358
Cat fur Female—TMT Female	0.531	0.282	55	1.883	0.5627
Cat body Female—Control Male	-0.111	0.236	55	-0.471	0.9998
Cat body Female—Cat body Male	1.580	0.415	55	3.808	0.0035
Cat body Female—Cat urine Male	-0.345	0.224	55	-1.538	0.7866
Cat body Female—TMT Male	0.231	0.258	55	0.894	0.9867
Cat urine Female—TMT Female	1.194	0.255	55	4.678	0.0001
Cat urine Female—Control Male	0.552	0.204	55	2.711	0.1191
Cat urine Female—Cat body Male	2.244	0.398	55	5.645	< .0001
Cat urine Female—Cat urine Male	0.318	0.190	55	1.679	0.7012
Cat urine Female—TMT Male	0.894	0.228	55	3.913	0.0023
TMT Female—Control Male	-0.642	0.276	55	-2.323	0.2806
TMT Female—Cat fur Male	1.050	0.439	55	2.391	0.2458
TMT Female—Cat urine Male	-0.875	0.266	55	-3.289	0.0224
TMT Female—TMT Male	-0.300	0.295	55	-1.017	0.9720
Control Male—Cat body Male	1.692	0.411	55	4.113	0.0010 *
Control Male—Cat urine Male	-0.234	0.217	55	-1.076	0.9619
Control Male—TMT Male	0.342	0.252	55	1.358	0.8762
Cat body Male—Cat urine Male	-1.925	0.405	55	-4.759	0.0001
Cat body Male—TMT Male	-1.350	0.424	55	-3.183	0.0316
Cat urine Male—TMT Male	0.575	0.241	55	2.392	0.2452

**Table A.6:** GLMM models with different distributions for the variable duration in the odour box per treatment for mice. All models included the experiment as a random factor.

	Treatment	Individual (random factor)	Distribution	AIC	df
M0	+		Binomial	608.841	9
M1	+	+	Binomial	272.053	10
M2 (Null)		+	Binomial	265.440	3

**Table A.7:** GLMM models with different distributions for the variable duration in odour box per treatment for rats. All models included the experiment as a random factor.

	Treatment	Individual (random factor)	Distribution	AIC	df
M0	+		Binomial	642.9638	9
M1	+	+	Binomial	467.8241	10
M1 Null		+	Binomial	463.2132	3

## Supporting information

S1 Data (XLSX)
